# Ethnic Differences in Disability Prevalence and Their Determinants Studied over a 20-Year Period: A Cohort Study

**DOI:** 10.1371/journal.pone.0045602

**Published:** 2012-09-28

**Authors:** Emily D. Williams, Therese Tillin, Peter Whincup, Nita G. Forouhi, Nishi Chaturvedi

**Affiliations:** 1 International Centre for Circulatory Health, Imperial College London, London, United Kingdom; 2 Division of Population Health Sciences and Education, St George's University of London, London, United Kingdom; 3 MRC Epidemiology Unit, Institute of Metabolic Science, Addenbrooke's Hospital, Cambridge, United Kingdom; Tehran University of Medical Sciences, Islamic Republic of Iran

## Abstract

**Background:**

To compare disability prevalence rates in the major ethnic groups in the UK and understand the risk factors contributing to differences identified. It was hypothesised that Indian Asian and African Caribbean people would experience higher rates of disability compared with Europeans.

**Methods:**

Data was collected from 888 European, 636 Indian Asian and 265 African Caribbean men and women, aged 58–88 years at 20-year follow-up of community-based cohort study, based in West London. Disability was measured using a performance-based locomotor function test and self-reported questionnaires on functional limitation, and instrumental (IADL) and basic activities of daily living (ADL).

**Results:**

The mean (SD) age of participants at follow-up was 69.6 (6.2) years. Compared with Europeans, Indian Asian people were significantly more likely to experience all of the disability outcomes than Europeans; this persisted after adjustment for socioeconomic, behavioural, adiposity and chronic disease risk factors measured at baseline (locomotor dysfunction: adjusted odds ratio (OR) 2.20, 95% CI 1.56–3.11; functional limitation: OR 2.77, 2.01–3.81; IADL impairment: OR 3.12, 2.20–4.41; ADL impairment: OR 1.58, 1.11–2.24). In contrast, a modest excess risk of disability was observed in African Caribbeans, which was abolished after adjustment (e.g. locomotor dysfunction: OR 1.37, 0.90–1.91); indeed a reduced risk of ADL impairment appeared after multivariable adjustment (OR from 0.99, 0.68–1.45 to 0.59, 0.38–0.93), compared with Europeans.

**Conclusions:**

Substantially elevated risk of disability was observed among Indian Asian participants, unexplained by known factors. A greater understanding of determinants of disability and normative functional beliefs of healthy aging is required in this population to inform intervention efforts to prevent disability.

## Introduction

As life expectancy increases, it is important to both quantify and understand determinants of ageing-related disability, such as functional limitations. Reports on trends in disability are inconsistent, with US studies suggesting significant declines over time [1], while some UK statistics indicate that the prevalence of severe disability may be rising [2]. There are marked socioeconomic differences; in the UK, people in the lowest socioeconomic group experienced an increase in disability between 1995 and 2001, while socially advantaged groups experienced a decline [2]. Ethnicity may play an independent role. In the US, there is evidence that African American people consistently experience significantly greater risk of disability, compared with White Americans [3]–[7]. Socioeconomic disparities explain a substantial proportion of this elevated risk [4]–[7], with health behaviours and chronic disease burden also playing a mediating role [4], [6].

People of Indian Asian and African Caribbean descent form the UK's two largest minority ethnic groups. First generation migrants arrived in the 1950 s and 60 s, and are now of pensionable age, when disability is a concern. Despite established health differentials between ethnic groups in the UK, there has been a lack of research exploring disability rates across British ethnic groups. Indian Asian people experience higher rates of coronary heart disease than Europeans [8], and both Indian Asian and African Caribbean groups show elevated risk of type 2 diabetes compared with Europeans [9], [10]. Other established risk factors for the development of disability, such as socioeconomic disadvantage and unhealthy behaviour profiles, are also known to vary across UK ethnic groups [11]–[14], and yet it is not known whether these variations in risk factors and rates of chronic disease predict ethnic group differentials in disability. If rates of disability do vary across ethnic groups, this is likely to have an inequitable impact on morbidity, quality of life and the economic burden on healthcare systems.

**Table 1:** Follow-up assessment of disability.

	Variables	Measurement/categorisation
**Clinic visit**		
Objective disability	Locomotor function – ‘Up and Go’ test [55], standardised measure of functional leg strength, power, and balance. Incorporates basic mobility movements needed for successful ageing.	Timed test involved participants getting up from a chair, walking three metres, turning around, walking and sitting back down; the threshold of ≥12 seconds was used to classify locomotor dysfunction [56], [57].
**Questionnaire**		
Self-reported disability	Functional limitations – “restrictions in performing fundamental physical activities” and are thought to be part of the pathway between risk factors andthe development of disability [38].	Impairment recorded if participants reported limitation with ≥1 of following:1) Walking unaided without stopping and discomfort; 2) walking up and down a flight of 12 stairs without resting; 3) bending down to pick up a shoe from the floor.
	Impairment of instrumental activities of daily living (IADL) – “needed for ‘independent living’ in society”.	1) Doing light housework; 2) shopping for personal items; 3) preparing one's own meals; 4) using the telephone; 5) taking medications; 6) managing money; 7) using public transport.
	Impairment of activities of daily living (ADLs) – activities “necessary for survival” [38].	1) Walking across a room; 2) getting in and out of bed; 3) getting in and out of a chair; 4) dressing and undressing oneself; 5) bathing or showering; 6) self-feeding; 7) getting to and using the toilet.

We hypothesised that, based on socioeconomic disadvantage and increased chronic disease risk, both minority ethnic groups would experience elevated rates of disability compared with Europeans.

## Methods

The Southall and Brent REvisited (SABRE) study is a tri-ethnic (European, Indian Asian and African Caribbean) 20-year community-based cohort recruited in West London between 1988 and 1991 [15]. Participants were aged 40–69 years at baseline, and the total available sample included 4857 (75% male) people of European (n = 2346), Indian Asian (n = 1710), and African Caribbean (n = 801) ethnic origin. Ethnicity was interviewer-recorded based on parental origin and appearance and subsequently confirmed by participants. A follow-up investigation of all surviving participants was performed between 2008 and 2011, 20 years after the baseline survey, when participants were aged 58–88 years.

### Ethics statement

All participants gave written informed consent. Approval for the study at baseline was obtained from Ealing, Hounslow and Spelthorne, and University College London research ethics committees, and at follow-up from St Mary's Hospital Research Ethics Committee (ref.07/H0712/109).

Following an overnight fast, the standard cardiometabolic assessments were performed, the protocol for which has been described elsewhere in detail [15]–[17]. A self-administered questionnaire included items on socioeconomic position (SEP – education, occupational grade [18], and home tenure), health behaviours (smoking, physical activity, total weekly alcohol intake, and sedentary behaviour), and medical history. A four-category indicator of life-course SEP was created with education (<11 versus ≥11 years) and occupational grade (manual versus non-manual) variables: low childhood/adult SEP; high childhood/low adult SEP; low childhood/high adult SEP; high childhood/adult SEP. Disability at baseline was assessed by questions concerning activity-limiting disability; this was dichotomised into those with or without disability at baseline.

### Follow-up assessments

Clinic attendees completed a similar questionnaire to baseline, and underwent a series of comprehensive clinical measurements [15]. Participants who could not attend were invited to complete a questionnaire, and were offered a home visit. Diabetes during the follow-up period was identified from medical record, participant recall of diagnosis, or follow-up OGTT, and CHD was identified by data extracted from primary care records [15]. Pain at follow-up was assessed using the relevant item from the EuroQol five-item health status (EQ-5D) scale [19]. Disability was measured using the objective ‘Up and Go’ test, as well as functional limitation, instrumental (IADLs) and basic activities of daily living (ADLs) scales (see table 1).

Of the original sample, 91% were traced, of whom 3333 participants were alive at follow-up. Questionnaire/clinical follow-up data were available for 2023 participants (978 European, 739 Indian Asian, and 306 African Caribbean), with follow-up response rates for questionnaire data of 60% in Europeans, 59% in Indian Asians, and 60% in African Caribbeans among traced survivors from the original sample (see Appendix 1 for participant flow diagram).

### Statistical analyses

Age- and sex-adjusted analyses of covariance and logistic regression were used, as appropriate, to compare the baseline (1988–1991) characteristics of responders (people who provided follow-up data) with non-responders (traced survivors who did not participate in follow-up). Subsequent analyses included only those people with complete questionnaire data (n = 1789, for locomotor function analyses n = 1292). Baseline characteristics were stratified by sex and compared across ethnic groups (Europeans as reference category), using chi-square tests, independent samples t-tests, and Mann Whitney U-tests as relevant.

**Figure 1:**
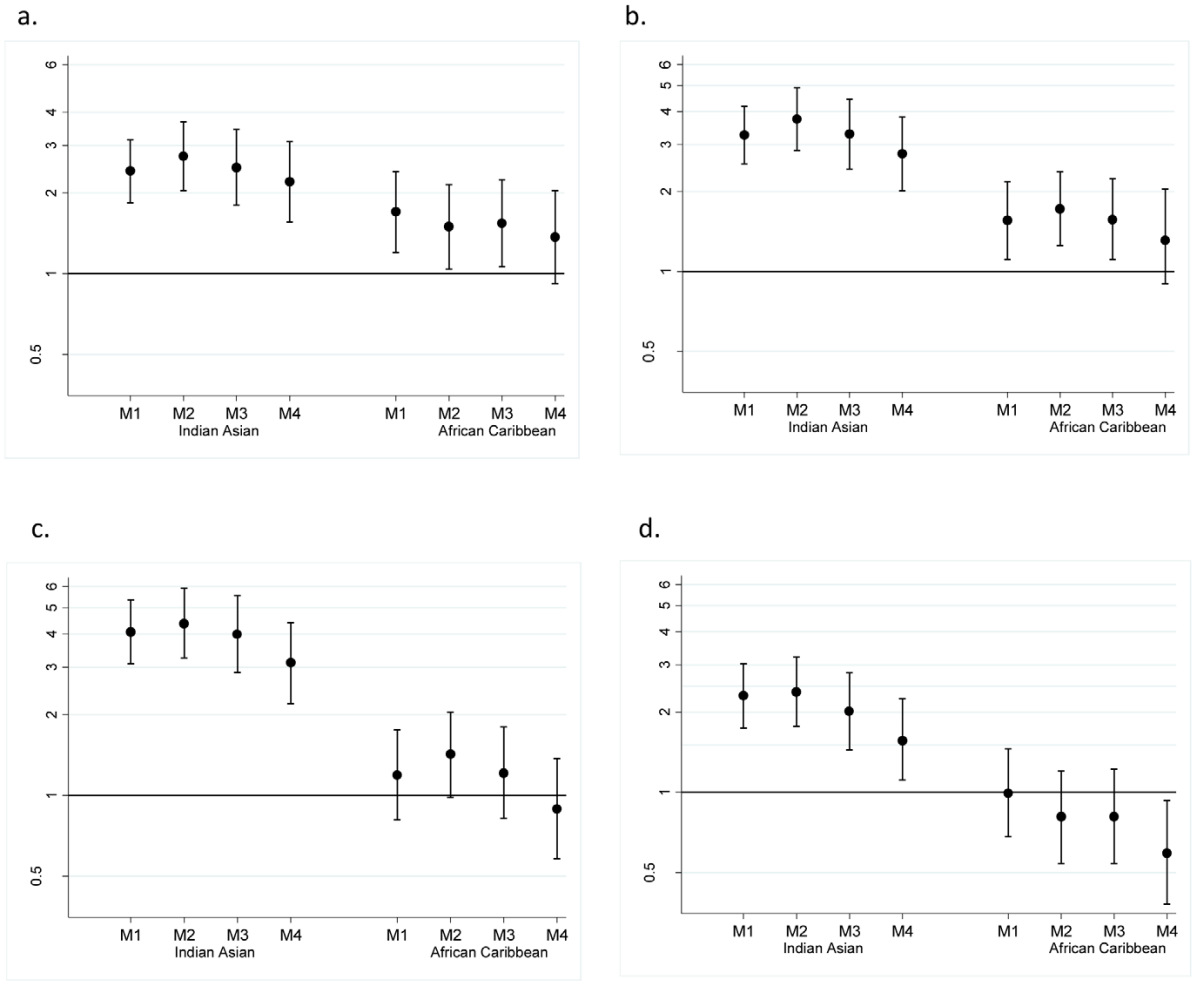
Risk of performance-based and self-reported disability, according to ethnic group. a: Risk of locomotor dysfunction. b: Risk of functional limitations c: Risk of an impairment of instrumental activities of daily living d: Risk of an impairment of activities of daily living.

Logistic regression analyses explored ethnic differences in locomotor dysfunction, functional limitations, IADL and ADL impairment (figure 1). We tested the models' fit using Hosmer and Lemeshow`s goodness-of-fit tests for each outcome. A range of sensitivity analyses were conducted to test the robustness of findings. All analyses were performed using SPSS version 18. Validation of the questionnaires was undertaken, the methods and results of which are available in Appendix 2.

## Results

### Comparison of baseline characteristics between responders and non-responders at follow-up

The proportions of responders (participants providing some data from traceable survivors) were 65%, 63%, and 63% among Europeans, Indian Asians and African Caribbeans, respectively. Differences between responders (n = 2132) and non-responders (n = 1204) were similar by ethnicity. Non-responders were older (*p*<0.001), more likely to be female (*p* = 0.003) and of lower SEP (based on education (70% of non-responders reported ≤11 years of education, compared with 62% among responders, *p*<0.001) and manual occupation (71% of non-responders compared with 63% of responders, *p*<0.001)). There were no group differences in physical activity levels, alcohol consumption, adiposity, prevalence of CHD, diabetes, or baseline disability after adjustment for age and sex.

### Baseline characteristics

By design [15], the majority of participants were male (table 2). At baseline, Indian Asian participants were younger than Europeans (*p*<0.001) while African Caribbean men were older (*p* = 0.007). Europeans were more likely to be in non-manual occupations, though Indian Asians and African Caribbean women reported more years of education. Europeans were also more likely to report their general state of health as good or very good. Behavioural profiles were mixed, with Europeans more likely to smoke, consume alcohol, and report more sedentary behaviour, while also performing higher levels of physical activity. Diabetes and hypertension were generally more frequent in the ethnic minority groups, but there were no differences in self-reported disability.

**Table 2:** Baseline characteristics by sex and ethnic group: SABRE study 1988–1991.

	European men (n = 689)	Indian Asian men (n = 552)	African Caribbean men (n = 142)	European women (n = 199)	Indian Asian women (n = 84)	African Caribbean women (n = 123)
Age	50.6 (6.4)	49.3 (6.0)*	52.6 (5.7)*	51.0 (6.5)	47.7 (5.6)*	51.2 (6.0)
Marital status – Married	83%	97%*	78%*	71%	87%*	57%*
Years lived in UK†	31.0 (1.0)	22.6 (6.3)*	29.8 (4.8)	33.2 (11.0)	21.1 (4.8)*	29.7 (5.6)
Years of education	11.1(2.7)	12.8 (3.7)*	10.9 (2.2)	10.6 (2.8)	11.0 (3.6)	11.2 (3.5)
Occupation – Manual labour	54%	73%*	86%*	46%	64%*	61%*
Home tenure – Own home	88%	93%*	74%*	80%	96%*	65%*
Life-course socioeconomic position (SEP)						
Low childhood/adult SEP	36%	20%	45%	35%	37%	36%
High childhood/low adult SEP	17%	53%	40%	11%	27%	26%
Low childhood/high adult SEP	15%	3%	4%	17%	8%	11%
High childhood/adult SEP	32%	25%*	11%*	37%	27%*	27%*
Smoking status – Current smoker	24%	12%	26%	23%	1%	8%
Ex-smoker	40%	11%	20%	25%	1%	9%
Never smoked	36%	77%*	54%*	53%	98%*	83%*
Physical activity (megajoules/week)	11.0 (7.5–16.5)	9.5 (6.0–13.0)*	11.0 (7.3–15.4)*	9.0 (5.2–13.4)	6.3 (2.0-9.8)*	10.0 (7.7–14.1)
Alcohol consumption (units/week)	12.1 (3.3–24.1)	3.1 (0–13.5)*	9.3 (2.2–23.3)	1.6 (0.2-6.2)	0*	0.8 (0.1–3.1)*
Sedentary behaviour (hours/week)	3.8 (1.0)	3.1 (1.1)*	3.1 (1.0)*	3.8 (1.0)	3.2 (1.0)*	3.2 (1.2)*
Body mass index (kg/m^2^)	26.0 (3.6)	25.5 (3.2)*	26.3 (3.0)	25.7 (4.6)	26.8 (4.6)	29.2 (5.1)*
Waist circumference (cm)	90.9 (10.3)	91.8 (9.3)	88.5 (8.9)*	78.5 (11.6)	83.2 (10.2)*	87.3 (11.7)*
Muscle mass (cm^2^)– Mid upper arm	64.9 (11.0)	60.5 (9.5)*	72.9 (11.8)*	44.5 (11.1)	37.6 (10.4)*	56.2 (12.0)*
Mid thigh	234 (34)	222 (32)*	252 (36)*	212 (37)	211 (40)	258 (44)*
Self-rated health – Very good/good	78%	65%*	67%*	70%	48%*	50%*
Coronary heart disease	4%	4%	2%	1%	0%	3%
Diabetes	5%	14%*	18%*	4%	8%	20%*
Hypertension	17%	27%*	39%*	16%	14%	49%*
Arthritis	12%	11%	13%	19%	20%	32%*
Asthma	9%	11%	7%	12%	7%	13%*
Disability	21%	21%	23%	28%	27%	33%

Data presented are unadjusted means (SD) and %, with exception of physical activity and alcohol consumption, presented as medians (interquartile range), due to skewed data (categorical variables were used for ethnic group comparisons). *p<0.05 for group differences with Europeans as reference category. †n = 959, includes only those people born outside the UK/Ireland with complete data (for European group, n = 61). Physical activity measured in megajoules expended per week during leisure time, travel time and sports. Sedentary behaviour measured as television viewing hours per week.

### Ethnic differences in disability at 58–88 years

#### Locomotor dysfunction

Prevalence of locomotor dysfunction in older age was 31% in Europeans, 46% in Indian Asians (*p*<0.001), and 49% in African Caribbeans (*p*<0.001) (table 3). Determinants of locomotor dysfunction included age, female sex, baseline SEP, self-rated health, chronic disease and central adiposity (table 4). There was the suggestion of an interaction between diabetes and ethnicity (*p* = 0.20 for interaction) on locomotor dysfunction, where diabetes appeared to have a greater effect in the Indian Asian group (odds ratio (OR) 3.12, 95% CI 1.65, 5.88, *p*<0.001), than in Europeans (OR 1.32, 0.50–3.53, *p* = 0.58). The Indian Asian excess in locomotor dysfunction (figure 1a) was accentuated upon adjustment for socioeconomic factors (model 2), being 2.7 fold greater than in Europeans. Adjustment for health behaviours, adiposity and chronic disease prevalence (model 4) only partially attenuated this Indian Asian vulnerability (OR 2.20, 1.56–3.11, *p*<0.001).

**Table 3:** Prevalence (n (%)) of disability outcomes across ethnic groups: SABRE study 2008–2011.

	European (n = 689)	Indian Asian (n = 552)	p value	African Caribbean (n = 265)	p value	European (n = 199)	Indian Asian (n = 84)	p value	African Caribbeans (n = 123)	p value
	Men					Women				
Performance-based measure										
Locomotor dysfunction*	141 (29)	179 (44)	<0.001	57 (49)	<0.001	53 (39)	32 (56)	0.031	51 (46)	0.32
Questionnaire-based measures										
Functional limitation	107 (16)	198 (36)	<0.001	43 (27)	0.001	56 (28)	35 (42)	0.026	57 (40)	0.02
Impairment of instrumental activities of daily living	68 (10)	172 (31)	<0.001	27 (17)	0.008	45 (23)	29 (35)	0.037	41 (30)	0.15
Impairment of activities of daily living	79 (12)	119 (22)	<0.001	19 (12)	0.82	47 (12)	29 (35)	0.059	35 (25)	0.77

Data presented as n (%).*Obtained in clinic attendees only: European men/women, n = 489/135, Indian Asian men/women n = 404/57, African Caribbean men/women n = 106/101. p values represent results from comparison with Europeans using χ2 tests.

**Table 4:** Associations between baseline risk factors and locomotor dysfunction at follow-up (age- and sex-adjusted) by ethnic group: Logistic regression analysis in the SABRE study.

	Risk of locomotor dysfunction		
	European	Indian Asian	African Caribbean
Age (per year)	1.15 (1.12–1.19)	1.08 (1.04–1.11)	1.13 (1.07–1.19)
Sex – Female	1.70 (1.10–2.61)	1.84 (1.04–3.27)	1.27 (0.71–2.28)
Life-course SEP–Low childhood/adult	1	1	1
High childhood/low adult	1.03 (0.59	0.55 (0.33	1.02 (0.49
Low childhood/high adult	0.69 (0.38	0.74 (0.26	0.69 (0.21
High childhood/adult	0.59 (0.38	0.70 (0.39	0.40 (0.17
Home tenure – Own home	0.39 (0.23–0.66)	0.26 (0.10–0.66)	0.59 (0.32–1.12)
Smoking status – Current smoker	1.49 (0.96–2.33)	1.02 (0.53–1.93)	1.09 (0.50–2.37)
Physical activity (quartiles) – Lowest	1	1	1
2	0.69 (0.41	0.93 (0.57	0.62 (0.27
3	0.44 (0.25	0.56 (0.33	0.68 (0.29
Highest	0.42 (0.25	0.79 (0.45	0.65 (0.27
Alcohol intake – Low	1	1	1
Moderate	0.45 (0.26	1.05 (0.62	0.68 (0.32
High	0.54 (0.34	1.34 (0.86	1.72 (0.78
Sedentary behaviour	1.12 (0.93–1.35)	0.93 (0.77–1.11)	1.04 (0.80–1.35)
Waist circumference (per cm)	1.05 (1.03–1.07)	1.04 (1.02–1.06)	1.03 (1.00–1.06)
Body mass index (per unit)	1.13 (1.08–1.19)	1.10 (1.04–1.17)	1.09 (1.02–1.18)
Self-rated health – Fair/ poor	1.49 (1.20–1.86)	1.30 (1.06–1.61)	1.87 (1.33–2.63)
Coronary heart disease	3.29 (1.19–9.07)	3.05 (0.94–9.90)	1.09 (0.22–5.56)
Diabetes	1.32 (0.50–3.53)	3.12 (1.65–5.88)	0.65 (0.27–1.54)
Hypertension	1.42 (0.87–2.33)	1.40 (0.90–2.17)	1.22 (0.67–2.23)
Arthritis	1.15 (0.67–1.98)	1.24 (0.67–2.29)	1.77 (0.88–3.58)
Asthma	0.50 (0.22–1.11)	1.67 (0.91–3.08)	1.49 (0.61–3.63)
Disability	2.03 (1.32–3.12)	1.82 (1.14–2.92)	1.89 (0.96-3.73)

Data presented as odds ratios (95% confidence intervals). Only includes people with complete questionnaire and locomotor function data (n = 1292). SEP: Socioeconomic position. Age, sedentary behaviour, waist circumference, and body mass index coded as continuous variables. Sex, life-course SEP (reference category: Low childhood and low adult), home tenure (reference category: Do not own home), smoking status (reference category: Never/ex-smoker), physical activity (megajoules per week categorised into quartiles, reference category: Lowest), alcohol intake (reference category: Low), self-rated health (reference category: Very good/good), and baseline coronary heart disease, diabetes, hypertension, arthritis, asthma and disability (reference category: No prevalent condition) coded as categorical variables.

African Caribbean participants also had an elevated risk of locomotor dysfunction (figure 1a), but with the inclusion of SEP, adiposity, and chronic disease, this ethnic difference was substantially attenuated (from OR 1.70, 1.20–2.40, *p* = 0.003, to OR 1.37, 0.92–2.04, *p = *0.13).

#### Functional limitations

Reported functional limitation was greater in Indian Asian (37%, *p*<0.001) and African Caribbean (32%, *p*<0.001) participants than Europeans (18%) (table 3). Similar baseline determinants were associated with reported functional limitation at follow-up as observed for locomotor dysfunction (data not shown). The Indian Asian excess in functional limitations was enhanced by adjustment for SEP (figure 1b), increasing the excess risk to over 3.5 fold compared with Europeans (OR 3.74, 2.85–4.92, *p*<0.001), and was not explained by health behaviours or chronic disease at baseline. In contrast, the excess risk reported for functional limitation among African Caribbean people was reduced, and rendered statistically non-significant after adjustment for SEP, adiposity, and chronic disease prevalence (OR 1.31, 0.90–1.91, *p* = 0.16).

#### IADL impairment

A significant excess in impairment of IADLs was observed in the Indian Asian group (table 2), with a greater than 4 fold excess when SEP was taken into account (figure 1c). Health behaviours and chronic disease appeared to explain some of the excess IADL impairment, however, after full adjustment, the Indian Asian group remained over three times more likely, than their European counterparts, to experience this disability outcome at follow-up (OR 3.12, 2.20–4.41, *p*<0.001).

Conversely, African Caribbean and European participants did not differ in their odds of IADL impairment (OR 0.89, 0.58–1.37, *p* = 0.59).

#### ADL impairment

Strikingly, while Indian Asians had higher levels of ADL impairment at follow-up (OR 1.58, 1.11–2.24, *p* = 0.011), prevalence in African Caribbeans was lower than in Europeans after multivariable adjustment (OR 0.59, 0.38–0.93, *p = *0.021) (figure 1d).

None of the goodness-of-fit tests showed statistically significant results.

#### Sensitivity analyses

Baseline muscle mass and pain (measured at follow-up) were included in alternative versions of the final model; these did not affect the ethnic group differences in the disability outcomes observed (data not shown).

‘Incident’ disability was explored by including only those people free from disability at baseline in the analyses (n = 1385); the same profiles of disability risk were observed across ethnic groups.

Models 1–4 were also repeated using the outcomes of major functional limitations and major IADL and ADL impairment, to explore differences in the extent of disability. We observed similar ethnic group differences in disability risk.

Analyses stratified by follow-up chronic disease status tested the possibility that underlying but undiagnosed (at baseline) chronic disease were driving the observed group differences. The same patterns of excess disability risk among Indian Asians were seen as observed previously.

The main analyses were completed in men only (numbers too small to perform in women only) to verify that results were not driven by female characteristics that differed between groups (sex interactions were non-significant); the ethnic group differences in disability remained.

### Questionnaire validation

Differential item functioning analyses identified item bias for two IADL and two ADL items for the Indian Asian group. Refinement of the scales, through removal of these items, replicated the patterns of excess impairment of IADL and ADL risk among Indian Asians observed previously (Appendix 2 for more detail).

## Discussion

In this tri-ethnic population in the United Kingdom, we observed a marked excess risk of disability in older age among the Indian Asian group, being two to four times higher, depending on the measure, compared with their European counterparts. This excess was observed using both objectively observed and self-reported measures, and could not be fully explained by SEP, health behaviours, co-morbidity, and body size measures in middle age. In contrast, people of African Caribbean descent had similar, or, after multivariable adjustment, lower levels of severe disability (ADL) compared with Europeans.

This is the first examination of disability in the main three ethnic groups in the UK using longitudinal data. The inclusion of performance- and questionnaire-based disability measurement, from mild physical dysfunction to more severe disability, and control for a wide range of covariates measured in middle age, are considerable strengths of this study.

Although studies have investigated ethnic differences in disability in other countries [3]–[7], [20], there is a dearth of literature examining disability among the UK's major ethnic groups. Our longitudinal finding of excess disability among Indian Asians is supported by one cross-sectional study from Singapore and one from the UK [20], [21].

No other data are available investigating the disability risk among UK African Caribbean people, however numerous studies from the US have reported an elevated risk of disability among African American people compared with White Americans [3]–[7], [22]–[24]. Despite the difference in national versus privatised healthcare systems in the UK and US, our findings are in line with those from African American samples. Most (but not all [4]) of this work shows that the majority of this excess risk is explained by known risk factors [7], [22], [24], and, in fact, also similar to our results, non-Hispanic Black groups have been shown to demonstrate reduced risk of certain functional outcomes, compared with Europeans, after multivariable adjustment [5], [6]. The similarity in our findings with the US, where socioeconomically deprived African Americans have been particularly disadvantaged by the private healthcare system [25], suggests that access to healthcare does not have a strong influence on disability rates between Black and White groups.

The current analyses examined the risk factors that explained the ethnic differences observed in disability, to identify intervention opportunities to reduce ethnic inequalities in disability. Inclusion of socioeconomic characteristics accentuated rather than attenuated the excess risk among Indian Asians. Although substantial literature documents greater socioeconomic disadvantage among UK minority ethnic groups [11], [26]–[28], this can depend on the SEP marker included (as shown by the divergent ethnic group patterns across the different socioeconomic characteristics here). Furthermore, the Indian Asian participants in our sample were predominantly Punjabi Sikh, one of the more advantaged South Asian subgroups in the UK [27]. Socioeconomic factors did explain some of the ethnic group differences between European and African Caribbean participants, supporting findings from African American groups [6], [7], [22], [29].

Health behaviours, adiposity, and chronic disease burden explained a small amount of the ethnic differences in disability among Indian Asian and African Caribbeans. Variations in behaviours are well established between European, Indian Asian and African Caribbean groups in the UK [13], [14], [27]. Variations in body composition [30], [31] and the relationship between fat distribution and disease outcomes in different groups [32], [33] mean that adiposity may have a differential impact on physical functioning across ethnicities. Inconsistent evidence exists regarding the role of muscle mass in the development of disability [34], [35], with some work suggesting that fat mass plays a stronger role [35], [36]. Our adjustment for a proxy marker of muscle mass did not affect the ethnic inequalities in disability in this study. Chronic diseases, such as CHD, diabetes, hypertension, and arthritis, are established predictors of disability development [37], [38], and our findings confirm previous work that chronic disease exposure explains a proportion of ethnic inequalities in disability [6], [24], [39]. Self-rated health was included in the models to capture any unmeasured chronic morbidity [40]; this, along with stratified models by follow-up chronic disease status (in sensitivity analyses) suggest that unmeasured chronic disease were not fully explaining the ethnic group differences observed.

The remaining excess risk among Indian Asians after full adjustment indicates that other factors explain these ethnic inequalities in disability. General factors associated with migration are unlikely to have influenced disability risk in this sample, since both the Indian Asian and African Caribbean groups comprised first generation immigrants. English was not the native tongue of the Indian Asian participants, and, although available language assistance was offered, residual difficulty may have contributed to the reported impairments of certain IADLs where English language proficiency is more salient, such as money-management and public transport use. It is possible that aspects of early life in South Asia or elements unique to the Indian Asian migration experience might have contributed to their increased disability risk. Possible examples include nutritional deficiencies and related conditions, such as osteomalacia and anaemia, at different life-course stages that may affect physical limitations [41], [42]. Although not unique to Indian Asian people, there is evidence that these deficiencies are more common in this ethnic group [43]–[45]. Another risk factor for disability [46], [47], Vitamin D insufficiency, is more common among UK Indian Asians and has been associated with pain levels in this group [48]. Although adjustment for pain at follow-up did not explain the disability risk here, future research should investigate the role of these factors in the excess disability risk among UK Indian Asians. Monitoring physical functioning in UK-born Indian Asians will distinguish the relative influence of early life/migration factors versus characteristics specific to their ethnic group. Although we did not have data on access to healthcare, it is unlikely that this would have significantly influenced the ethnic differences observed, since UK Indian Asians make equitable use of healthcare services [49].

Although the IADL scales have been validated in Indian groups [50], a systematic difference in interpretation of or response to questionnaires by ethnicity may remain [51]. The validation performed here identified item bias in some of the IADL and ADL items, yet their removal did not affect the observed results. Thus, although interpretation of questions may vary across ethnic groups and English language facility may affect interaction with life outside the home, these factors should not influence the performance-based measure of locomotor dysfunction. Nonetheless, cultural differences in perceptions and expectations of healthy ageing may still influence the way Indian Asians respond to disability performance tests and questionnaires [21], [52], [53], for example, responses to activity tasks and scales could be based more on expectations of functional capacity or suitability for certain tasks rather than on actual physical capabilities. Therefore, our results could exhibit a false inflation of disability differentials between Indian Asians and Europeans. Due to cultural and family norms, functioning ‘dependence’ may not reflect the same reduction in quality of life among South Asians, as observed in other groups [54]. Future research should examine whether disability has an equivalent impact on quality of life, social functioning, and other morbidity outcomes across elderly Indian Asian and other ethnic groups.

Other limitations should be considered. The loss to follow-up means that the group is likely to be subject to attrition bias. The Indian Asians in this sample were at a lower risk of dying than Europeans (unpublished data) and therefore survival bias is unlikely to have affected the Indian Asian disability estimates observed. Further, when assessing burden of disability in older age, individuals by definition must have survived until then. Although health and socioeconomic response gradients existed at baseline, they were consistent across groups, and with no baseline differences in disability levels, it is unlikely this bias played a major role in our findings. The self-report baseline behavioural data introduces possible measurement error, which may have caused an imprecise estimation of the mediating/confounding role of covariates. Although socioeconomic disadvantage was assessed in multiple ways, residual effects of other aspects of SEP (such as wealth and income) may contribute to the results observed. The analyses presented here consider Indian Asians as a single group but we must recognise the subgroup heterogeneity, in terms of SEP, CHD incidence and risk profiles [11], [26]. The expression, Indian Asian, was used because the majority of the sub-sample was born in India, with approximately half of Punjabi Sikh ethnicity. With no information available on visual and hearing ability, the contribution of these impairments towards the outcomes studied cannot be ruled out.

The unexplained excess disability among older Indian Asian people in the UK observed in this study has substantial health, wellbeing and socioeconomic implications for these groups. Furthermore, provision of the governmental disability living allowance is based on items from the questionnaires used in this study, and therefore the disparities observed could contribute to significant economic burden as the UK's elderly population grows. A greater understanding of both the determinants and expectations of physical functioning in older age is required. This would inform the timing and choice of therapeutic interventions to directly address these inequalities, but may also encourage the development of educational strategies to promote realistic norms of healthy ageing across all cultures.

## Supporting Information

Appendix S1 **Figure of participant flow diagram.**
(TIF)

Appendix S2 **Validation of self-reported disability questionnaires.**
(DOCX)
