# Supplementary figures and images for: Ethnic Differences in Disability Prevalence and Their Determinants Studied over a 20-Year Period: A Cohort Study
Source: PLoS One. 2012 Sep 28;7(9):e45602. doi: 10.1371/journal.pone.0045602 (PMC3460991; doi:10.1371/journal.pone.0045602)

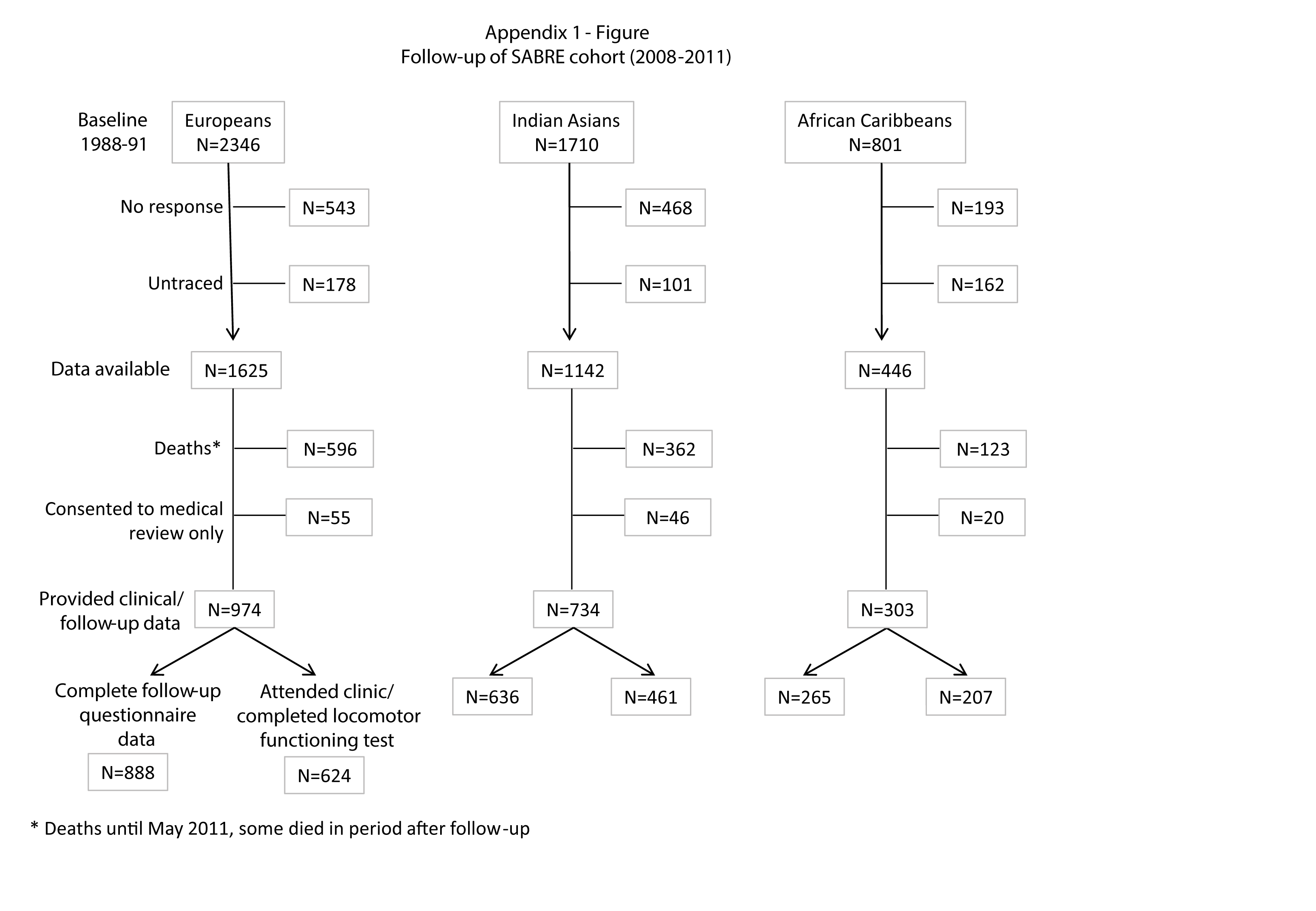

Supplement: Appendix S1 — Figure of participant flow diagram. (TIF) [file pone.0045602.s001.tif]
