# Supplementary material for: Ethnic Differences in Disability Prevalence and Their Determinants Studied over a 20-Year Period: A Cohort Study
Source: PLoS One. 2012 Sep 28;7(9):e45602. doi: 10.1371/journal.pone.0045602 (PMC3460991; doi:10.1371/journal.pone.0045602)
Supplement: Appendix S2 — Validation of self-reported disability questionnaires. (DOCX) [file pone.0045602.s002.docx]

**Appendix 2**

**Validation of self-reported disability questionnaires**

**METHODS**

**Statistical analyses**

To test the construct validity for the use of self-reported disability scales across the ethnic groups, Spearman’s correlations were performed between performance-based locomotor function and the self-reported outcomes (functional limitations, IADLs and ADLs), all modelled as continuous variables. These correlations were conducted on the full sample and separately by ethnic group. Differential item functioning (DIF) analyses were also administered on each of the self-report functioning outcomes to determine whether item bias was present between ethnic groups (with the European group as the reference category). The process used to measure DIF was the Mantel Chi-Square values (Mantel) and the Standardised Liu-Agresti Cumulative Common Log-odds ratio values (LOR Z). Mantel scores greater than 6.63 indicated a rejection of the null hypothesis that the different groups responded in the same way on the studied items [58]. LOR Z values greater than 2.0 or less than -2.0 were indicative of DIF effect size. For the identification of DIF, non-parametric tests within the DIFAS 4.0 program were used ([1](#_ENREF_1)). Refined versions of the scales, i.e. scales no longer displaying DIF, were created by removing the DIF items. To test whether previously observed ethnic group differences were still present, fully-adjusted logistic regressions (Model 4) were re-analysed using the refined self-report outcomes.

**RESULTS**

*Construct validity*

Table 1 displays the correlation coefficients for the whole sample and divided by ethnic group. The correlation between the performance-based and self-report measures does not appear to substantially differ across the ethnic groups.

**Table 1: Correlation between performance-based and self-reported disability outcomes**

|  | **Functional limitations** | **IADLs** | **ADLs** |
| --- | --- | --- | --- |
| All | 0.36 | 0.35 | 0.37 |
| European | 0.32 | 0.28 | 0.33 |
| Indian Asian | 0.31 | 0.39 | 0.36 |
| African Caribbean | 0.39 | 0.27 | 0.45 |

Data presented as Spearman’s correlation coefficients. IADLs: Instrumental Activities of Daily Living. ADLs: Activities of Daily Living. All presented correlations represent p < 0.001.

*Content validity*

There was no evidence of item bias in the functional limitation scale across either Indian Asian or African Caribbean responses, with Europeans as the reference category. For the IADL scale, two items displayed signs of item bias (Mantel scores > 6.63, LOR Z scores > 2) among the Indian Asian group (regarding housework and use of public transport). Using a refined version of the IADL scale (omitting the DIF items), the same patterns of IADL impairment were observed across Indian Asian (OR 4.60, 95% CI 2.88-7.35) and African Caribbean groups (OR 0.46, 0.23-0.92). With regards to the ADL scale, there was no sign of item bias for the African Caribbean group, however two items demonstrated item bias among the Indian Asian group (bathing and toilet use). With these items removed, a refined ADL impairment variable was tested and the same ethnic differences were observed (Indian Asians: OR 2.75, 1.55-4.89; African Caribbeans: OR 0.71, 0.35-1.46).

**References:**

1. Penfeld R. DIFAS 4.0: Differential item functioning analysis system: user's manual. 2007.
